# Tangerine Peel Polysaccharide Alleviates High-Fat Diet-Induced Renal Lipotoxicity in Association with Gut Microbiota Remodeling in Mice

**DOI:** 10.3390/ijms27198824

**Published:** 2026-10-02

**Authors:** Jingqiu Ma, Xiuyuan Wu, Chunbo Wu, Rihui Wu

**Affiliations:** 1 College of Food Science and Engineering, Lingnan Normal University, Zhanjiang 524048, China; 2 School of Pharmacy and Food Engineering, Wuyi University, Jiangmen 529020, China

**Keywords:** tangerine peel, polysaccharide, gut microbiota, renal lipotoxicity, chronic kidney disease

## Abstract

Chronic kidney disease (CKD) may result from ectopic fat accumulation induced by a high-fat diet (HFD), which has been closely linked to dysbiosis of the gut microbiota. In this study, we investigated the effects of a polysaccharide fraction derived from tangerine peel, Pericarpium *Citri Reticulatae* “Chachiensis” (PCRCPI), on renal fat accumulation and lipotoxicity in mouse models. Oral administration of PCRCPI markedly reduced renal fat deposition, inflammation, fibrogenesis and dysfunction in HFD-fed mice. PCRCPI treatment notably upregulated the mRNA expression of growth differentiation factor 15 (*GDF-15*), a stress-response molecule, and downregulated lipid synthesis in the kidney. PCRCPI reshaped the HFD-induced intestinal microbial composition and structure by enriching beneficial bacteria, including *Ligilactobacillus* and *Lactobacillus*. Transplantation with gut microbiota from PCRCPI-treated mice effectively upregulated *GDF15* mRNA expression and alleviated renal dysfunction and lipotoxicity in HFD-fed mice, indicating that the effects of PCRCPI in maintaining *GDF-15* expression and preventing renal lipotoxicity may be dependent on the gut microbiota. This study introduces a potential prophylactic treatment that may act through gut microibota-associated pathways and offers valuable insights into the mechanisms of CKD prevention.

## 1. Introduction

Chronic kidney disease (CKD) is recognized as a distinct risk factor for end-stage renal disease, marked by permanent alterations in renal function and morphology. The worldwide prevalence of CKD is escalating swiftly, and its associated mortality ranks as the seventh leading cause of death globally, representing a considerable challenge to public health [1]. The incidence of CKD is primarily attributed to genetic factors, dietary modifications, and comorbid conditions, including obesity and diabetes mellitus [2,3]. Obesity induced by a high-fat diet is a significant risk factor for both the onset and progression of kidney disease, posing a substantial challenge to the effective management of CKD individuals [4]. Therefore, the development of innovative, highly effective, and more tolerable pharmacological agents aimed at addressing the underlying mechanisms of CKD is critically important for the management of the kidney damage induced by a high-fat diet (HFD).

Extensive clinical and animal evidence indicates that prolonged HFD intake promotes obesity and renal lipotoxicity [5]. These conditions are closely linked to gut dysbiosis, characterized by altered microbial composition and diversity in HFD-fed rodents [6,7]. Mechanistically, excess intestinal lipids modify microbial growth conditions, favoring specific bacterial populations. HFD-induced dysbiosis impairs bile acid metabolism and intestinal barrier integrity, and increases translocation of bacterial products such as lipopolysaccharide (LPS). LPS activates Toll-like receptor 4 (TLR4) signaling, provoking inflammatory responses and oxidative stress that sustain gut dysbiosis and hasten CKD progression [8]. In line with this, CKD patients exhibit reduced microbial diversity and decreases in beneficial taxa with concomitant enrichment of pathogenic taxa [9,10]. These microbial community shifts can modulate host signaling pathways. For instance, certain microbes like *Akkermansia muciniphila* can induce growth differentiation factor 15 (GDF-15), a stress-responsive member of the TGF-β superfamily and a pivotal regulator of metabolic disease [11]. In renal tissue, GDF15 inhibits TLR4–NF-κB signaling and ferroptosis, mitigates lipid accumulation and injury, and serves as a predictor of CKD progression [12]. Collectively, these data indicate that GDF15 is a plausible mechanistic conduit linking the gut microbiota to renal lipotoxicity. However, it remains unknown whether specific microbes regulate GDF15 to attenuate renal lipotoxicity.

Dietary modification, particularly polysaccharide supplementation, has been regarded as the most effective strategy for reestablishing gut microbiota homeostasis and improving obesity-related phenotypes [13]. The varied sources of polysaccharides from traditional Chinese medicine result in differences in sugar composition and molecular structures [14], which may act as specific prebiotics. Pericarpium *Citri Reticulatae* ‘Chachiensis’ (PCRC), commonly known as Chenpi in Chinese or tangerine peel in English, is derived from the dried mature fruit peel of *Citrus reticulata Blanco*. This traditional medicinal herb has been used in Asian countries for millennia and is recognized for its cardiovascular protective effects [15]. Its chemical compounds, including flavonoids, volatile oils, and alkaloids, have been acknowledged as the primary bioactive constituents of CRP [16]. Notably, CRP extracts rich in polymethoxy flavonoids have emerged as potential protective agents against metabolic disorders and circulatory system diseases, attributed to their significant anti-inflammatory, antioxidant, and anti-apoptotic properties [17,18]. In practical applications, whether for medicinal, culinary, or tea purposes, the aqueous solution of tangerine peel is predominantly utilized, with water-soluble polysaccharides (PCRCPs) serving as the principal active components. In our previous studies, we found that oral administration of PCRCPs effectively mitigated obesity and intestinal inflammation while enhancing the populations of probiotic *Lactobacillus johnsonii* and *Parabacteroides goldsteinii* in HFD-fed and colitic mice, respectively [19,20]. This indicates that PCRCPs can modulate the gut microbiota across various pathological conditions, promoting beneficial bacterial growth and exerting protective effects. More importantly, we previously identified a polysaccharide fraction derived from PCRC (PCRCPI) as the most effective component against metabolic syndrome, which reshaped the gut microbiota composition and enriched *Lactobacillus* spp. in HFD-fed mice [21]. Nevertheless, the precise mechanisms and consequences of PCRCPI-induced gut microbiota modulation on renal lipotoxicity remain to be elucidated.

In light of these findings, we hypothesized that PCRCPI may alleviate HFD-induced renal lipotoxicity through modulation of the gut microbiota. To test this hypothesis, we investigated the impact of PCRCPI on renal lipotoxicity in an HFD-fed murine model and evaluated the contribution of gut microbiota using fecal microbiota transplantation (FMT).

## 2. Results and Discussion

### 2.1. Oral PCRCPI Administration Alleviated HFD-Induced Renal Injury

**Figure 1:**
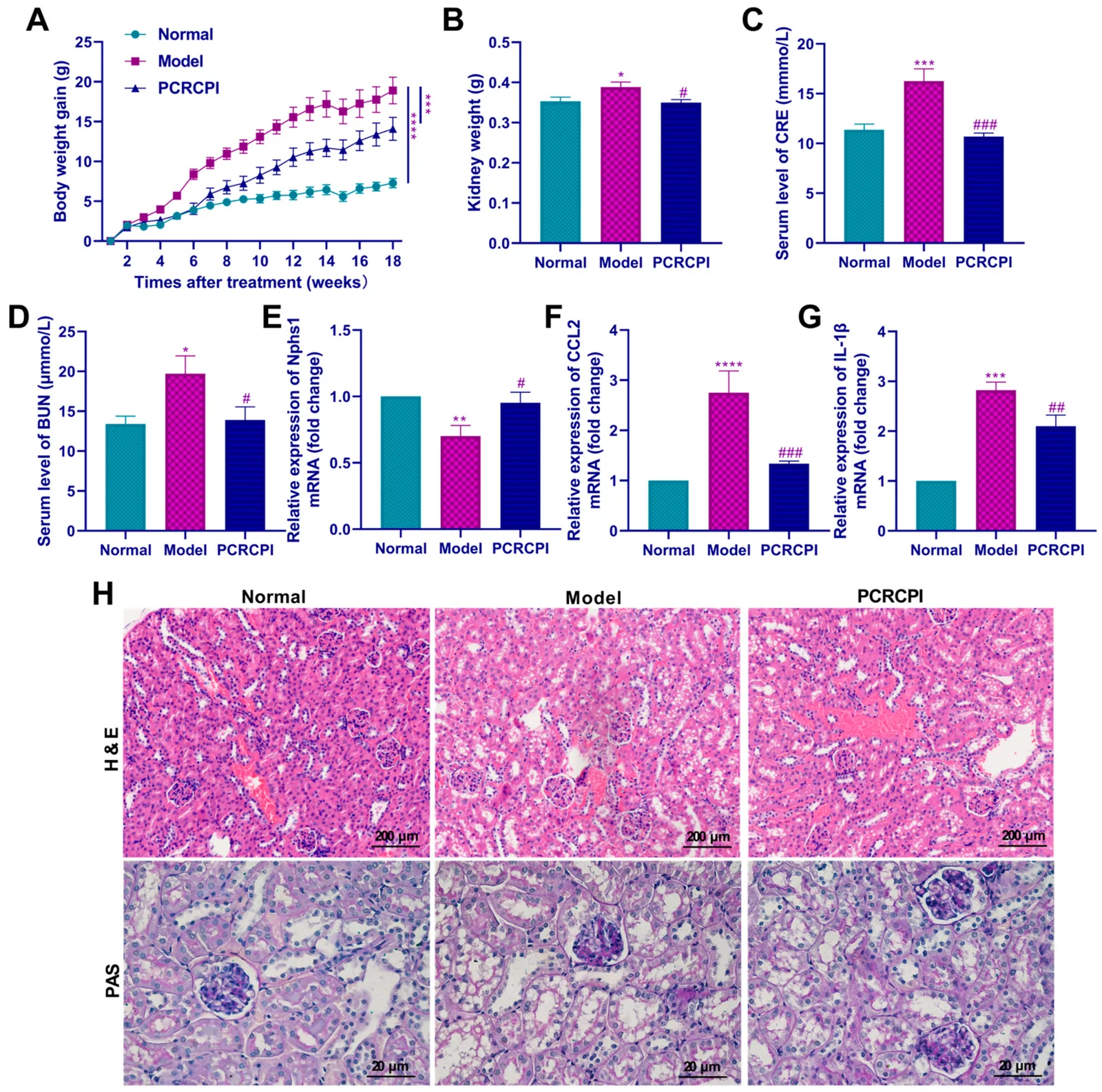
Oral administration of PCRCPI improved renal dysfunction and injury in HFD-fed mice. (**A**) Body weight gain during the experimental period. (**B**) Kidney weight. (**C**,**D**) The levels of TG and TC in serum, respectively. (**F**,**G**) Serum CRE and BUN levels. (**E**–**G**) Relative mRNA expression levels of *Nphs1*, *CCL2* and *IL-1β* in the kidneys. (**H**) Representative images of H&E and PAS staining of kidney tissues. Data are expressed as mean ± SEM (*n* = 8). * *p* < 0.01, ** *p* < 0.01, *** *p* < 0.001, **** *p* < 0.0001 versus the normal group. # *p* < 0.01, ## *p* < 0.01, ### *p* < 0.001 versus the model group.

High-fat and fructose-rich diets are widely recognized as major contributors to obesity and associated metabolic disorders, including CKD [22,23]. In the present study, HFD-fed mice were used a model of chronic kidney injury to evaluate whether oral PCRCPI administration could mitigate renal lipotoxicity. HFD-fed mice were administered PCRCPI at 60 mg/kg/d or were not treated for 18 weeks. Compared with mice fed a standard chow diet, HFD-fed mice exhibited significantly greater body weight gain and kidney weights, which increased by approximately 170.6% and 9.9%, respectively (Figure 1A,B). Compared with untreated HFD controls, PCRCPI administration not only significantly decreased food consumption (Figure S1), but also markedly decreased body weight gain and kidney weight in HFD-fed mice, with reductions of 33.2% and 10.0%, respectively (Figure 1A,B). HFD feeding also resulted in a pronounced increase in perirenal fat mass compared with the chow diet, an effect that was significantly attenuated by PCRCPI treatment (Figure 1C). Moreover, serum urea and creatinine levels were markedly elevated in HFD-induced obese mice compared with normal controls; however, PCRCPI treatment effectively suppressed these HFD-induced increases (Figure 1C,D). PCRCPI consumption markedly restored renal nephrotic syndrome 1 (*Nphs1*) mRNA expression in HFD-fed mice, with a 35% increase compared to untreated HFD controls (Figure 1E). *Nphs1* encodes nephrin, a critical component of the glomerular filtration barrier and a marker associated with the risk of congenital nephrotic syndrome [24]. In addition, PCRCPI treatment significantly suppressed the HFD-induced upregulation of the mRNA expression of the pro-inflammatory cytokines C-C motif chemokine ligand 2 (*CCL2*) and interleukin-1β (*IL-1β*), with reductions of 35.3% and 25.5%, respectively (Figure 1F,G). Histological analysis using HE and PAS staining demonstrated tubular vacuolization, tubular epithelial injury, glomerular atrophy, and collagen fiber deposition in the tubular interstitium of HFD-fed mice (Figure 1H), indicating severe renal injury following prolonged HFD consumption. Notably, these pathological abnormalities were significantly ameliorated by PCRCPI intervention. Collectively, these findings indicate that PCRCPI administration exerted protective efficacy in mitigating renal lipotoxicity in HFD-induced obese mice. Nevertheless, the use of a single PCRCPI dose (60 mg/kg/d) constitutes a limitation of this study because it precludes assessment of dose–response relationships. Whether lower doses provide comparable efficacy, higher doses confer additional benefits, or the response reaches a plateau remains to be established. Future studies incorporating multiple dosage levels are warranted to optimize the protective regimen and further characterize the dose-dependent renoprotective effects of PCRCPI.

### 2.2. Oral PCRCPI Increased Renal GDF-15 Expression and Suppressed Lipid Deposition and Renal Fibrosis in HFD-Induced Obese Mice

**Figure 2:**
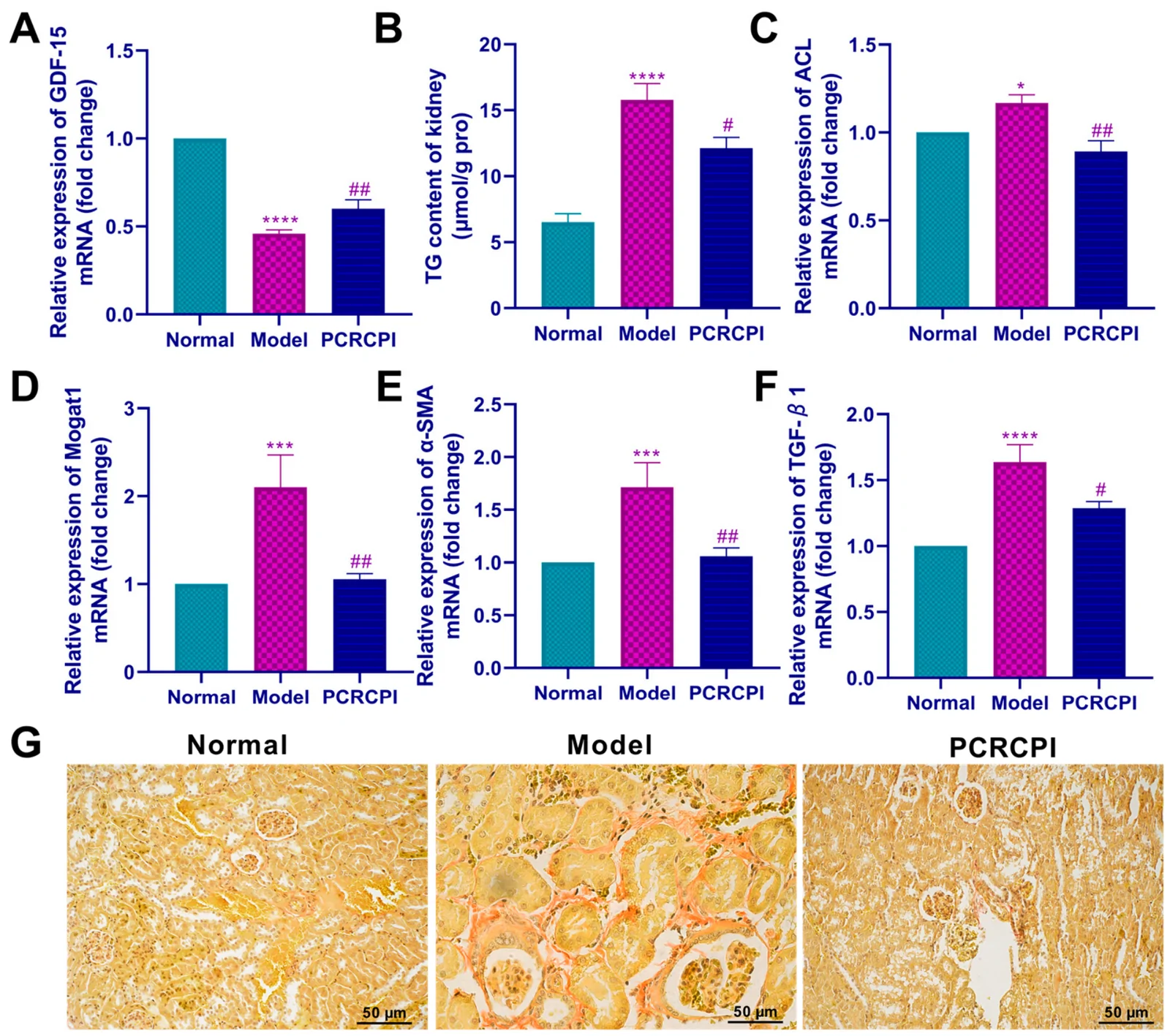
Treatment with PCRCPI inhibited renal lipogenesis and fibrosis in HFD-fed mice. (**A**) Relative mRNA expression of renal *GDF-15*. (**B**) The content of TG in kidney. (**C**,**D**) Relative mRNA expression of renal *ACL* and *Mogat1*, respectively. (**E**,**F**) Relative mRNA expression of renal *α-SMA* and *TGF-β1*, respectively. (**G**) Representative images of Sirius red staining of kidney tissue. Data are expressed as mean ± SEM (*n* = 8). * *p* < 0.05, *** *p* < 0.001, **** *p* < 0.001 versus the normal group. ^#^ *p* < 0.01, ^##^ *p* < 0.01, versus the model group.

Given that GDF-15, a stress-, metabolism-, and inflammation-related factor, is notably reduced in the plasma, liver, and adipose tissue of obese and diabetic rodents [25,26] and may be downregulated in association with improvement of the gut-kidney axis, we examined whether renal *GDF-15* mRNA expression was altered in HFD-fed mice. We found that renal *GDF-15* mRNA expression was significantly reduced in HFD-fed mice compared to chow-fed controls (Figure 2A). Notably, PCRCPI treatment significantly upregulated renal *GDF-15* mRNA expression in HFD-fed mice by 30.4% relative to untreated HFD controls (Figure 2A), indicating that PCRCPI-induced upregulation of renal *GDF-15* may contribute to the reduction in renal lipid accumulation. This interpretation is consistent with previous evidence showing that GDF-15 overexpression reduces food intake and body weight in both rodents and humans [27,28]. As a stress-responsive cytokine implicated in energy homeostasis and appetite regulation, GDF-15 has been reported to protect against renal lipid accumulation and fibrosis, partly by suppressing lipogenic gene expression and inhibiting inflammatory cascades [27,29]. Consistent with the increase in *GDF-15* expression, PCRCPI treatment significantly reduced renal lipid accumulation, as evidenced by the lower renal TG levels and decreased mRNA expression of the lipid synthesis regulatory genes ATP-citrate lyase (*ACL*) and monoacylglycerol transferase 1 (*Mogat1*) in HFD-fed mice relative to untreated mice (Figure 2B–D). Moreover, PAS staining revealed tubular vacuolization and collagen fiber deposition in the glomerular and tubular interstitium of HFD-fed mice, both of which were markedly ameliorated by PCRCPI administration (Figure 2G and Figure S2). Consistent with these histopathological improvements, PCRCPI treatment significantly downregulated the renal mRNA expression of α-smooth muscle actin (*α-SMA*) and transforming growth factor-β (*TGF-β*) in HFD-fed mice (Figure 2E,F), both of which are fibrogenic factors involved in the development of renal fibrosis. Given that renal fibrosis is an inevitable consequence of excessive extracellular matrix accumulation and is characteristic of nearly all forms of chronic kidney disease [30], we propose that PCRCPI may possess the capacity to prevent HFD-induced renal fibrosis. However, future mechanistic studies are warranted to determine whether GDF-15 signaling is involved in the renoprotective effects of PCRCPI.

### 2.3. Oral PCRCPI Modulated Gut Microbiota Composition and Alleviated Gut Dysbiosis in Mice with Kidney Injury

**Figure 3:**
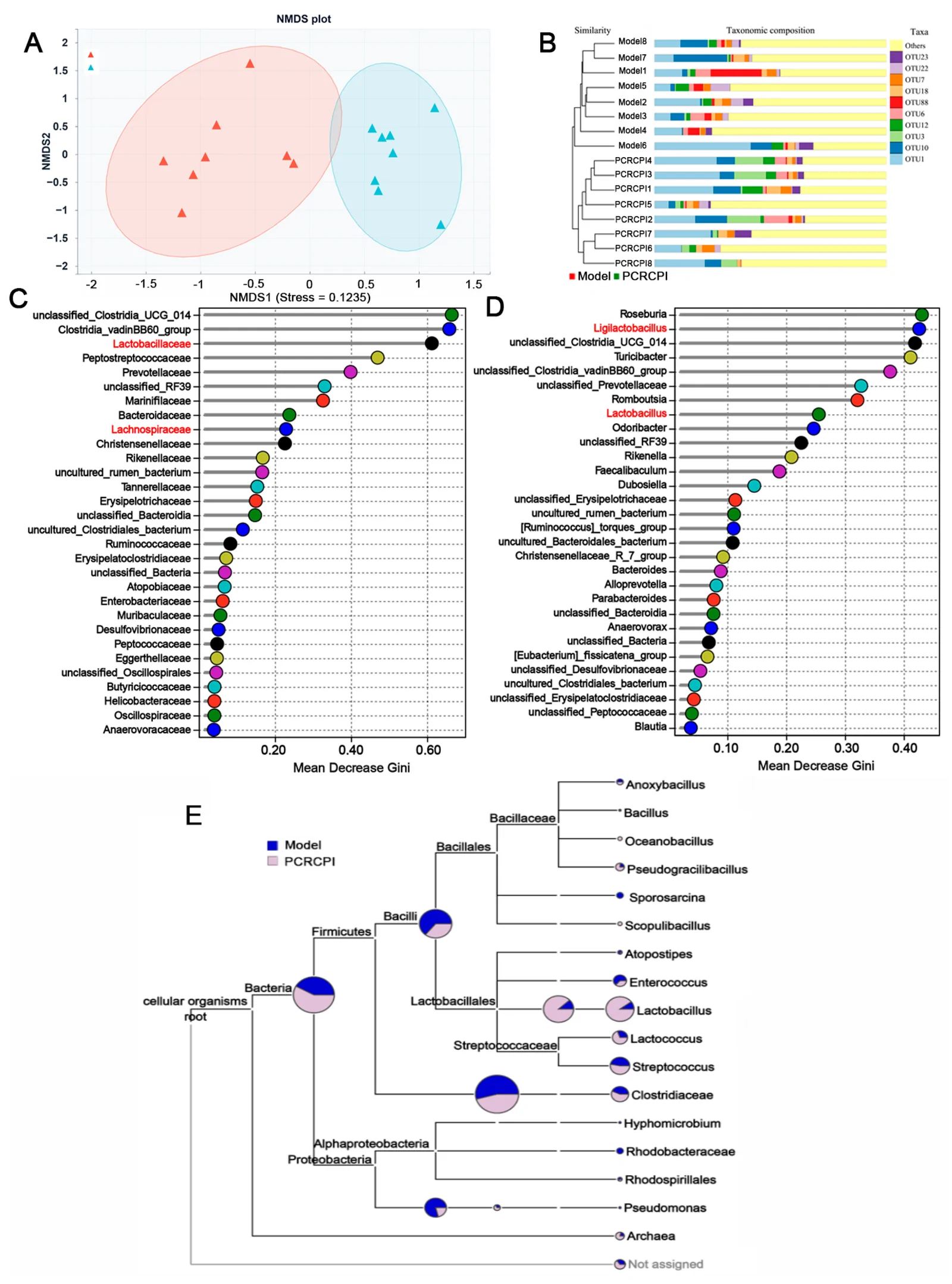
PCRCPI administration alleviated gut microbiota dysbiosis in mice with renal lipotoxicity. The gut microbiota of HFD-fed mice and mice administered 60 mg/kg/d PCRCPI was characterized by 16S rDNA pyrosequencing (*n* = 8 per group). (**A**) Non-metric multidimensional scaling (NMDS) based on OTU-level Binary-Jaccard dissimilarity. (**B**) Hierarchical clustering of gut microbiota using the unweighted pair-group method with arithmetic mean (UPGMA) based on Binary-Jaccard dissimilarity. (**C**,**D**) The top 30 bacterial taxa contributing to the mean decrease Gini measure in the random forest regression analysis at the family and genus levels, respectively. (**E**) MEGAN taxonomy tree illustrating taxa assigned to the microbial communities of the Model group (blue) and PCRCPI group (pink).

Given the reciprocal and specific interactions between gut microbiota and dietary polysaccharides [31,32], we were keen to explore how PCRCPI treatment influences the gut microbiota composition in HFD-fed mice with kidney injury. Fecal samples were subjected to 16S rRNA gene sequencing, as previously reported. PCRCPI treatment did not affect the alpha diversity of gut microbiota in mice with renal lipotoxicity (Figure S3), consistent with previous findings [21]. However, non-metric multidimensional scaling (NMDS) revealed clear separation of the overall microbial structures between the Model and PCRCPI groups (Figure 3A). Consistently, the unweighted pair-group method with arithmetic mean (UPGMA) clustering showed that Model samples clustered together and were distinctly separated from PCRCPI samples (Figure 3B), indicating substantial differences in gut microbial community composition between the two groups.

We next compared differential taxa between the two groups at multiple taxonomic levels using random forest analysis. At the family level, PCRCPI treatment significantly increased the relative abundances of *Lachnospiraceae* and *Lactobacillaceae*. At the genus level, PCRCPI-treated mice exhibited significantly higher relative abundances of *Lachnospiraceae_NK4A136*_group, *Ligilactobacillus*, and *Lactobacillus*, all of which are recognized as potentially beneficial bacteria that contribute to the maintenance of gut homeostasis (Figure 3C,D). For example, *Limosilactobacillus reuteri* and *Lactobacillus acidophilus* have been demonstrated to mitigate obesity-related disorders by correcting gut microbiota dysbiosis and improving intestinal permeability [33,34]. In line with previous Linear Discriminant Analysis Effect Size (LEfSe) analysis (>4), the relative abundances of *Lactobacillales*, *Lactobacillaceae*, *Ligilactobacillus*, and *Lactobacillus* were significantly higher in PCRCPI-treated mice than in untreated controls, whereas *Rhodobacteraceae* and *Proteobacteria* were markedly enriched in the Model group [21], as indicated by MEGAN taxonomic tree analysis (Figure 3E). *Proteobacteria* is generally regarded as an indicator of gut dysbiosis because it normally represents only a minor component of the healthy gut microbial community [35]. Collectively, these findings indicate that PCRCPI administration significantly reshaped the gut microbiota composition and increased GDF-15 expression in mice with HFD-induced renal lipotoxicity.

### 2.4. Transplantation of PCRCPI-Reshaped Gut Microbiota Prevented Renal Injury in HFD-Fed Mice

**Figure 4:**
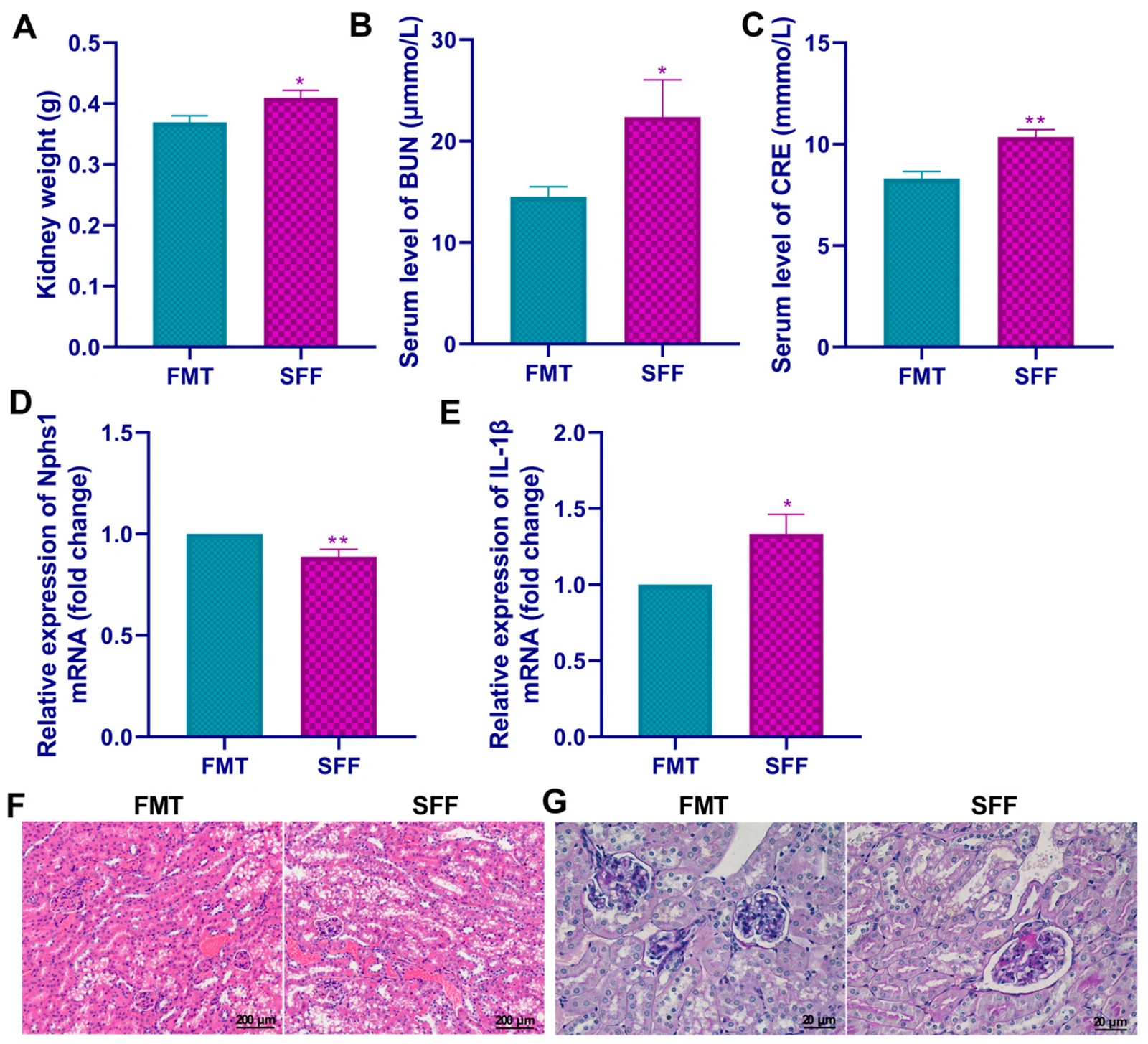
The PCRCPI-reshaped gut microbiota facilitated the protective effects against HFD-induced kidney injury. (**A**) Renal weight of the recipient mice. (**B**,**C**) Levels of BUN and CRE in serum, respectively. (**D**,**E**) Relative mRNA expression levels of *Nphs1* and *IL-1β* in the kidneys of the recipients, respectively. (**F**,**G**) Representative histological images of H&E and PAS staining in the kidneys of the recipients, respectively. Data are presented as mean ± SEM (*n* = 8). * *p* < 0.05, ** *p* < 0.01.

To further elucidate the beneficial effects of PCRCPI on gut microbiota-mediated renal lipotoxicity, fecal microbiota from mice receiving PCRCPI gavage were transferred to conventional recipient mice fed an HFD. After 15 weeks of colonization, recipient mice that received PCRCPI-conditioned microbiota (FMT group) exhibited a significant reduction in kidney weight compared with those receiving sterile fecal filtrate (SFF group), without affecting food intake (Figure 4A and Figure S4). Relative to SFF recipients, FMT treatment significantly decreased serum BUN and CRE levels (Figure 4B,C), and attenuated renal inflammation and injury, as evidenced by decreased *IL-1β* mRNA expression, increased *Nphs1* mRNA expression, and improved renal histomorphology according to H&E staining (Figure 4D–F). Moreover, FMT recipients displayed significantly attenuated renal fibrosis compared with SFF recipients (Figure 4G). Collectively, these findings demonstrate that transplantation of PCRCPI-reshaped gut microbiota may contribute to the prevention of renal injury and the preservation of renal function.

### 2.5. FMT Downregulated GDF-15 Expression and Prevented Renal Lipotoxicity in HFD-Fed Mice

**Figure 5:**
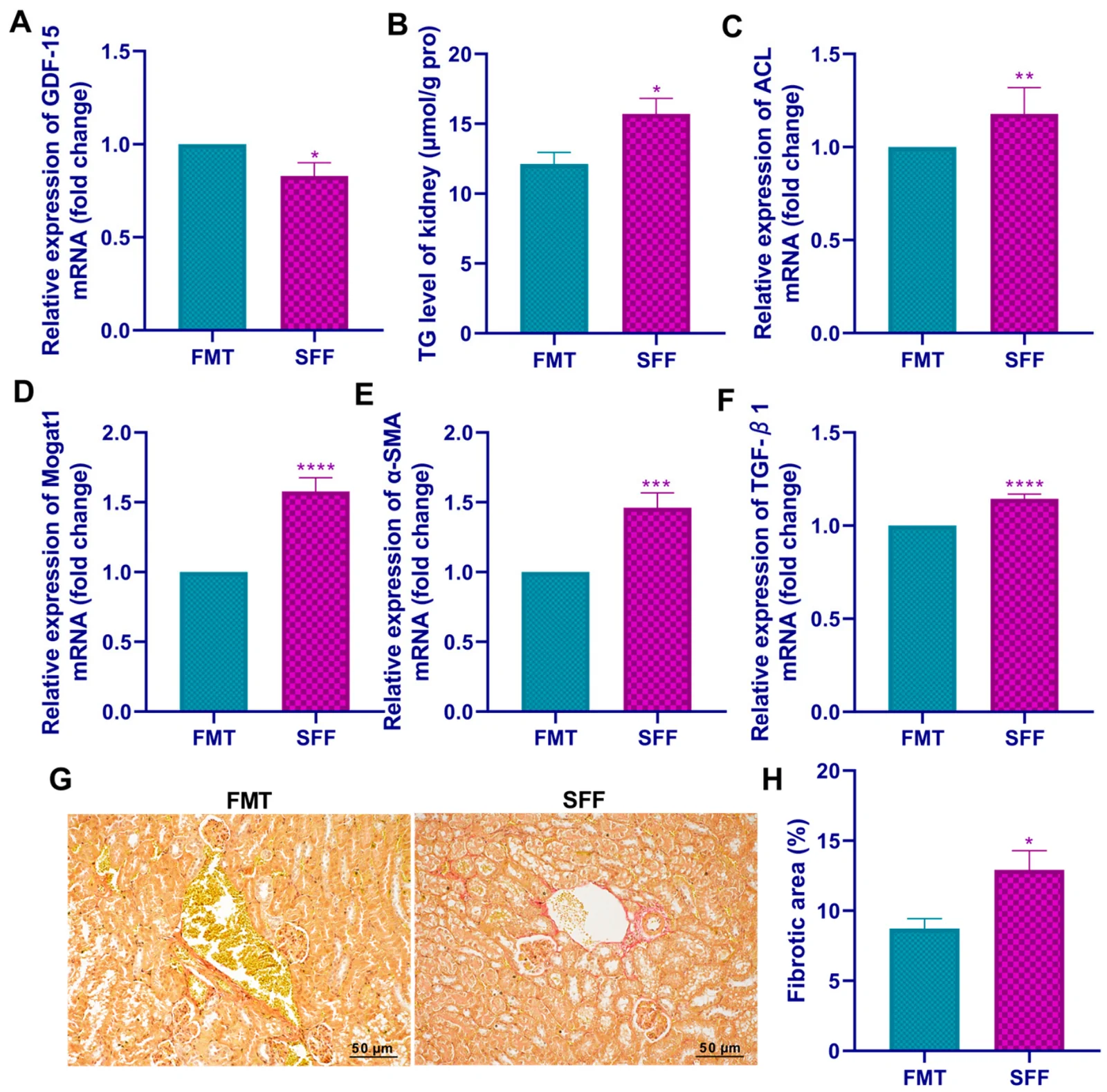
PCRCPI-reshaped gut microbiota preserved *GDF-15* expression and prevented renal lipotoxicity induced by an HFD. (**A**) Relative mRNA expression of renal *GDF-15* in the recipients. (**B**) TG content in the kidneys of the recipients. (**C**,**D**) Relative mRNA expression levels of renal *ACL* and *Mogat1* in the recipients, respectively. (**E**,**F**) Relative mRNA expression levels of renal *α-SMA* and *TGF-β1* in the recipients, respectively. (**G**) Representative histological images of Sirius red staining in the kidneys of the recipients. (**H**) Quantification of renal fibrosis by Sirius red staining (*n* = 4). Data are presented as mean ± SEM (*n* = 8). * *p* < 0.05, ** *p* < 0.01, *** *p* < 0.001, **** *p* < 0.0001.

To further confirm the effects of gut microbiota transferred from PCRCPI-treated mice on *GDF-15* expression and renal lipotoxicity in HFD-fed mice, we performed additional analyses. FMT recipients exhibited significantly higher renal *GDF-15* mRNA expression than SFF controls (Figure 5A), consistent with the increased *GDF-15* expression observed in PCRCPI-treated donor mice (Figure 2A). Moreover, FMT significantly reduced renal lipid accumulation, as indicated by the decreased TG content and lower mRNA expression of *ACL* and *Mogat1* in HFD-fed mice (Figure 5B–D). Notably, fecal microbiota transfer from PCRCPI-treated mice reduced the mRNA expression of renal fibrosis-related markers, including *α-SMA* and *TGF-β1*, and decreased renal collagen deposition, as shown by Sirius red staining, compared with SFF controls (Figure 5E–H), demonstrating that transplantation of fecal microbiota from PCRCPI-treated mice alleviated HFD-induced renal fibrosis. These findings suggest that horizontal fecal microbiota transfer from PCRCPI-treated mice conferred metabolic protective effects similar to those observed in PCRCPI-treated mice (Figure 4 and Figure 5). Thus, the PCRCPI-reshaped gut microbiota may contribute to the upregulation of *GDF-15* expression and the anti-lipotoxic effects of PCRCPI in preventing the development of HFD-induced kidney injury.

### 2.6. FMT Reproduced the PCRCPI-Associated Gut Microbiota Profile in HFD-Fed Mice

**Figure 6:**
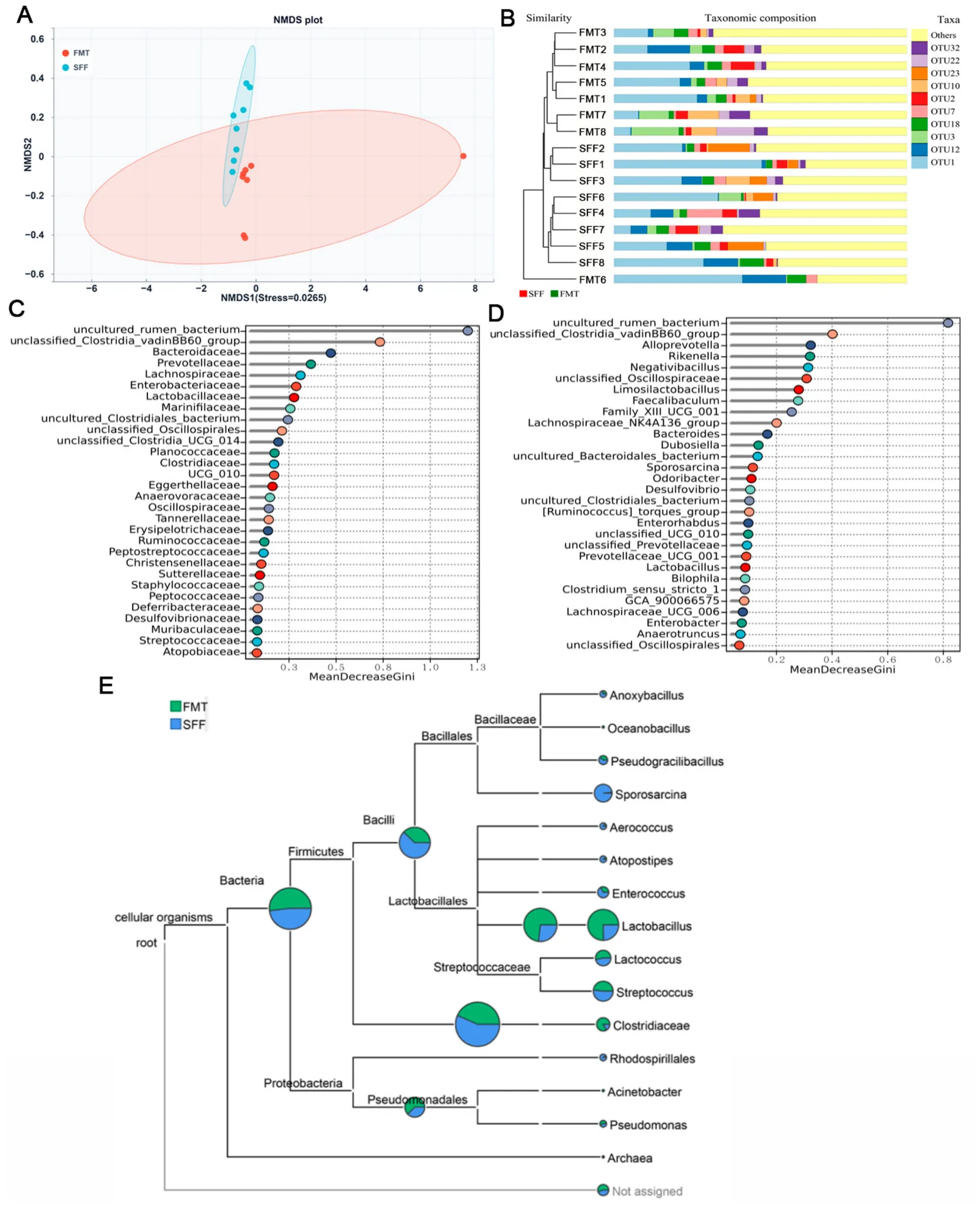
Alterations in gut microbiota composition in HFD-induced obese mice following fecal microbiota transplantation from PCRCPI-treated donors. (**A**) Non-metric multidimensional scaling (NMDS) analysis of recipient mice. (**B**) Analysis of UPGMA based on Binary-Jaccard dissimilarity. (**C**,**D**) The top 30 bacterial strains that contributed to the increase in the mean decrease Gini measure identified through random forest regression at both the family and genus levels, respectively. (**E**) The composition of microbial communities of the FMT group (Green) and SFF group (blue) shown as a MEGAN taxonomy tree.

To validate the colonization of PCRCPI-modified microbiota in mice exhibiting renal lipotoxicity, 16S rDNA sequencing was performed to characterize the intestinal bacterial composition of recipients. Consistent with the PCRCPI-treated mouse model, alpha diversity remained unchanged in HFD-fed mice receiving FMT from PCRCPI-exposed mice compared with control recipients (Figure S5), as described in our previous study [21]. NMDS analysis of beta diversity further revealed a distinct gut microbiota composition between the two recipient groups (Figure 6A). Consistent with these differences in beta diversity, UPGMA analysis showed pronounced divergence in microbial clustering between the FMT and SFF groups (Figure 6B). These findings indicate that FMT from PCRCPI-treated mice significantly altered the gut microbial composition of HFD-fed recipients.

Random forest analysis revealed that, at the family level, HFD-fed mice receiving FMT exhibited greater relative abundances of *Lachnospiraceae*, *Bacteroidaceae*, and *Lactobacillaceae* than mice receiving SFF (Figure 6C). At the genus level, fecal microbiota transfer from PCRCPI-treated significantly increased the relative abundances of *Ligilactobacillus, Bacteriodes*, and *Lactobacillus* compared with SFF recipients (Figure 6D). Notably, in line with the donors, *Ligilactobacillus* and *Lactobacillus* were among the top 30 enriched discriminative taxa in recipients of PCRCPI-modified microbiota (Figure 6C-E), partly reflecting the dominant microbial community structure and distribution of the donor. Collectively, these findings demonstrate that the gut microbiota may play a central role in PCRCPI-mediated metabolic protection.

## 3. Materials and Methods

### 3.1. Materials and Reagents

Five-year-old tangerine peel was obtained from XINHUI XINBAOTANG ORANGE PEEL Co., Ltd. (Jiangmen, Guangdong, China). Polysaccharides were extracted using a hot-water extraction and alcohol precipitation method, as previously reported [36,37,38]. Briefly, tangerine peel powder was refluxed with petroleum ether at 85 °C for 8 h in total (4 h per cycle, repeated twice) to remove lipids, followed by immersion in 95% ethanol for 4 h to eliminate impurities. The treated material was subsequently filtered, dried, and subjected to ultrasonic extraction with a defined volume of distilled water. Specifically, 5.0 g of sample was transferred to a conical flask and extracted three times under specified ultrasonic extraction time, power, and temperature conditions. The extracts were combined, centrifuged at 4000× *g* for 15 min, and concentrated using a rotary evaporator. Four volumes of 95% ethanol were then added, and the mixture was maintained at 4 °C for 12 h. The precipitate was collected by filtration and lyophilized to obtain tangerine peel polysaccharides. The polysaccharide content was quantified using the phenol-sulfuric acid method. Following purification of PCRCP fractions and characterization, the PCRCPI was identified as an acidic polysaccharide enriched with components D-galacturonic acid and arabinose, as described in our previous studies [20,21].

### 3.2. Animals

Male C57BL/6J mice, aged eight weeks and weighing 20.0 ± 2.0 g, were obtained from Zhuhai BesTest Bio-Tech Co., Ltd. (Zhuhai, China). The animals housed under specific pathogen-free conditions with a 12 h light/dark cycle and relative humidity maintained between 44% and 65%. Before initiation of the experiment, the mice were acclimatized for one week with ad libitum access to clean water and a standard diet. All animal welfare measures and experimental procedures were approved by the Animal Ethics Committee of International Institute for Healthcare Innovation (Jiangmen, China) (approval No. N2022028) and conducted in compliance with institutional and national guidelines for the care and use of laboratory animals.

### 3.3. Animal Experiments

A kidney-injured mouse model was established using a high-fat diet based on our previous obesity mouse model [21]. Prior to the study, a power analysis was performed to determine the required sample size. Assuming an effect size of a 30% reduction in key renal parameters (e.g., serum creatinine and renal triglyceride content), a standard deviation of 15%, a two-sided significance level of α = 0.05, and a statistical power of 80%, the minimum sample size was calculated to be 6 mice per group. To account for potential attrition during the long-term experiment, 8 mice per group were used.

Briefly, mice were randomly assigned to three groups, including a normal group, a model group, and a treatment group (*n* = 8 per group). Mice in the normal group were provided with a standard diet (10% fat, 3.5 kcal/g diet), while both the model and treatment groups were fed a high-fat diet (HFD, 45% fat, 16.8 kcal/g diet) for a duration of 18 weeks. Furthermore, mice in the treatment groups received PCRCPI at a dosage of 60 mg/kg/d via gavage for the entire 18-week period. Mice in the normal and model groups were administered an equivalent volume of vehicle by gavage. At the end of the animal experiment, fresh fecal samples, serum, and kidney tissues were collected for subsequent analyses.

### 3.4. Fecal Microbiota Transplantation (FMT)

**Figure 7:**
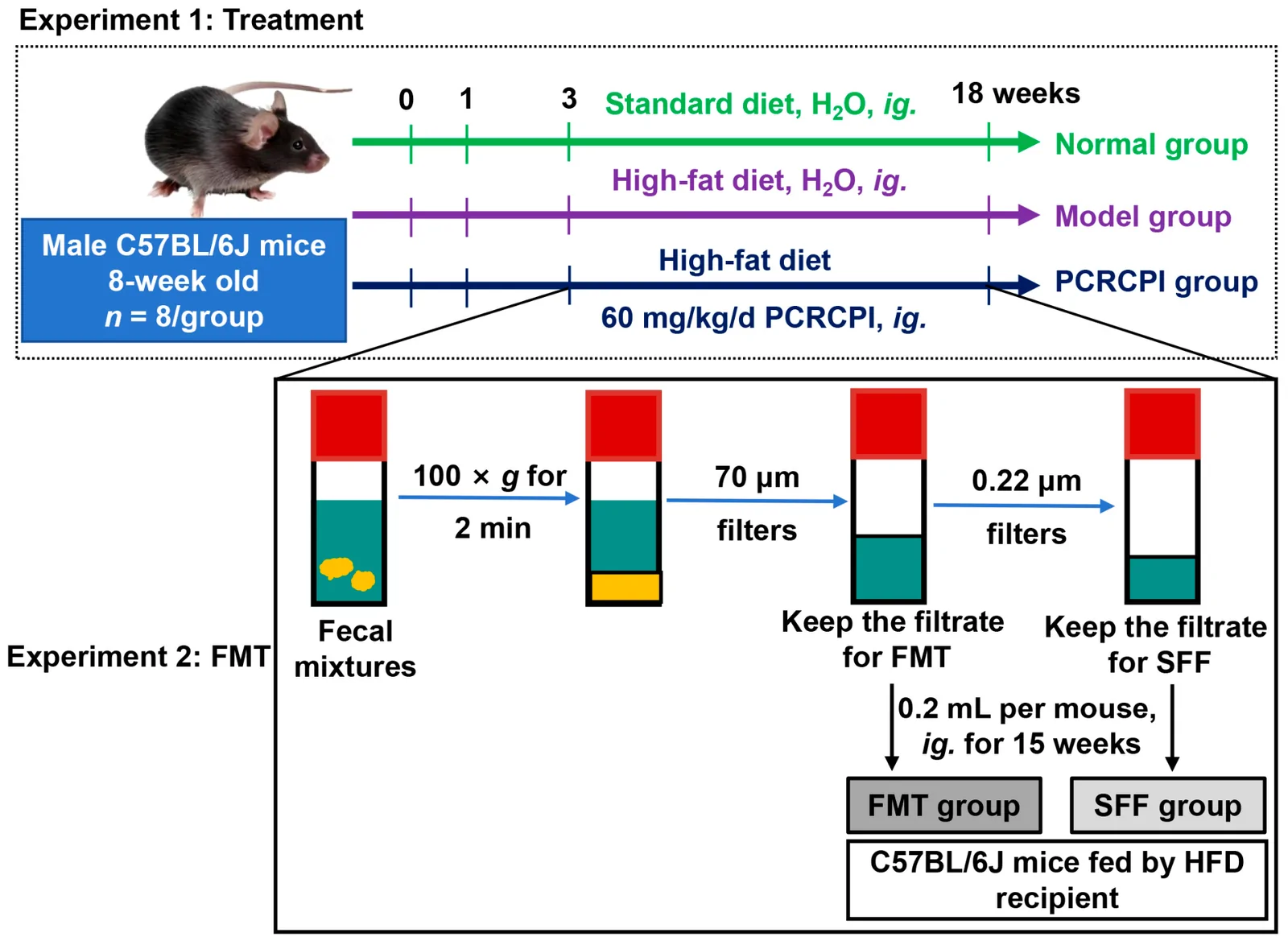
Schematic diagram of the experimental design. In Experiment 1, mice were divided into three groups and fed either a standard chow diet or an HFD. HFD-fed mice in the treatment group received PCRCPI (60 mg/kg/d) by oral gavage for 18 weeks. In Experiment 2, fresh fecal samples were collected from PCRCPI-treated donor mice beginning at week 3. The fecal suspensions were filtered sequentially through 70-μm and 0.22-μm filters to prepare the bacterial-containing inoculum (FMT) and the sterile filtrate (SFF), respectively. The resulting supernatants were orally administered once daily to HFD-fed recipient mice for 15 weeks.

For the FMT experiment, fresh fecal pellets were collected daily from PCRCPI-treated HFD donor mice (*n* = 8) starting from week 3 of the intervention period. The fresh collected feces were pooled, thoroughly homogenized in sterile saline to a final concentration of 100 mg feces/mL, and centrifuged at 100× *g* for 2 min to remove large debris. The resulting supernatant was then divided into two portions for separate preparation (Figure 7).

For the FMT group (*n* = 8), the supernatant was filtered through a 70-μm pore-size filter to remove residual particulate matter while retaining intact bacterial cells. This filtrate (0.2 mL per mouse) was administered daily by oral gavage to conventional HFD-fed recipient mice for 15 weeks.

For the SFF (sterile fecal filtrate) control group (*n* = 8), the supernatant was further filtered through a 0.22-μm pore-size filter to completely remove all bacteria. This sterile filtrate (0.2 mL per mouse) was administered daily by oral gavage to a separate group of conventional HFD-fed recipient mice for 15 weeks, serving as a placebo control to account for the effects of the gavage procedure and non-bacterial components of the fecal material.

### 3.5. Biochemical Analyses

Blood creatinine (CRE) and urea nitrogen (BUN) levels were assessed utilizing commercial detection kits from Nanjing Jiancheng Bioengineering Institute (Nanjing, China) in accordance with the manufacturer’s protocols. Triglyceride (TG) and total cholesterol (TC) concentrations in kidney tissue were quantified using assay kits from the same institute. Specifically, kidney tissue was combined with absolute ethanol at a 1:9 ratio of tissue weight (g) to ethanol volume (mL), followed by homogenization and centrifugation at 600× *g* for 10 min. The resulting supernatant was analyzed for absorbance at 510 nm using a microplate reader (Thermo Scientific, Waltham, MA, USA).

### 3.6. Histological Analysis

Fresh kidney specimens were immediately fixed in 4% paraformaldehyde for a minimum of 24 h and subsequently paraffin-embedded. The resulting tissue blocks were sectioned at a thickness of 5 μm, deparaffinized in xylene, and rehydrated through a graded ethanol series. Kidney sections were stained with hematoxylin and eosin (H&E), periodic acid-Schiff (PAS) and Sirius red reagent according to standard protocols [39,40]. Histological images were captured using an Olympus CX23 microscope (Tokyo, Japan). For quantitative analysis, five random fields per section were examined. Sirius Red-positive areas were quantified using ImageJ software (version 1.53; National Institutes of Health, Bethesda, MD, USA) and expressed as a percentage of the total field area. For each individual mouse, measurements from all sections and fields were first averaged to obtain a single animal-level value. These animal-level values were then used for statistical comparison between groups, with the individual mouse treated as the biological replicate. All histological assessments and quantifications were performed in a blinded manner by two independent observers.

### 3.7. Real-Time qPCR

Gene expression was analyzed by quantitative real-time PCR (qPCR) following a standard protocol [41]. Briefly, total RNA was extracted from kidney tissue using TRIzol reagent (Life Technologies, Carlsbad, CA, USA) according to the manufacturer’s instructions. RNA concentration and purity were assessed using a Nanodrop 2000 spectrophotometer (Thermo Fisher Scientific, Waltham, MA, USA), and RNA integrity was verified by 1.5% agarose gel electrophoresis. Equal amounts of total RNA (1 μg) were reverse-transcribed into complementary DNA (cDNA) using a PrimeScript RT reagent kit with gDNA Eraser (Vazyme, Nanjing, China) following the manufacturer’s protocol. Gene expression analysis was performed using a SYBR Green Premix Ex Taq II kit (Vazyme, Nanjing, China) on an ABI 7500 Real-Time PCR System (Applied Biosystems, Foster, CA, USA). The PCR cycling conditions were as follows: initial denaturation at 95 °C for 30 s, followed by 40 cycles of 95 °C for 5 s and 60 °C for 34 s, with a final dissociation stage to verify amplicon specificity. All reactions were performed in triplicate for each sample, and non-template controls were included in each run to confirm the absence of contamination. The mouse β-actin gene was validated as a stable internal reference across all experimental groups and was used to normalize target gene expression. Primer efficiency for each gene was evaluated using standard curves generated from serial dilutions of pooled cDNA, and all primers showed amplification efficiencies between 90% and 110%. Relative gene expression levels were calculated using the 2^−ΔΔCt^ method. The primer sequences used in this study are listed in Supplementary Table S1.

### 3.8. 16S rRNA Gene Sequencing

Genomic DNA was extracted from fecal samples using the TIANamp Stool DNA Kit (TianGen Biotech Co., Ltd., Beijing, China) according to the manufacturer’s instructions. DNA concentration was measured using a Nanodrop 2000 spectrophotometer (Thermo Fisher Scientific, Wilmington, DE, USA), and DNA integrity was assessed by 1.5% agarose gel electrophoresis. The full-length 16S rRNA gene was amplified using the 27F (AGRGTTYGATYMTGGCTCAG) and 1492R (RGYTACCTTGTTACGACTT) universal primer set. PCR products were purified and used for library construction with the PacBio SMRTbell Express Template Prep Kit V.3.0 (Pacific Biosciences, Menlo Park, CA, USA) following the manufacturer’s instructions. Sequencing was performed on the PacBio platform (Biomarker Technologies Co., Ltd., Beijing, China). Raw reads were quality-filtered and processed using QIIME2 (v.2019.4). Amplicon sequence variants (ASVs) were inferred using the DADA2 R Statistics package (v1.20.0). Representative sequences were taxonomically assigned based on the GreenGenes reference database (v.13.8). Alpha diversity was assessed using the Chao1 and Shannon indices. Beta diversity was evaluated using non-metric multidimensional scaling (NMDS) based on Binary-Jaccard distances, and random forest analysis was performed to identify taxa that discriminated between groups and to assess the significance of group separation. Data analysis and visualization were performed using the BMKCloud platform (www.biocloud.net).

### 3.9. Statistical Analysis

The acquired data are expressed as the mean ± standard error of the mean (SEM) and visualized using GraphPad Prism 8.0 software (GraphPad Software, San Diego, CA, USA). For comparisons between two groups, statistical significance was assessed by using a two-tailed Student’s t-test. In cases involving more than two groups were analyzed, a one-way analysis of variance (ANOVA) with Tukey’s post hoc test was employed, followed by multiple comparisons. *p*-values < 0.05 were deemed statistically significant.

## 4. Conclusions

In conclusion, this investigation demonstrated that oral administration of PCRCPI confers substantial renoprotective effects against HFD-induced lipid accumulation and renal injury, possibly through a gut microbiota-associated mechanism. PCRCPI treatment significantly increased renal *GDF-15* mRNA expression and corrected gut microbial dysbiosis. Fecal microbiota transplantation from PCRCPI-treated mice preserved mRNA expression and conferred significant protection against HFD-induced renal lipotoxicity. These findings reveal a potential mechanism underlying the protective effects of PCRCPI and identify a promising protective treatment for HFD-induced renal lipotoxicity.

## Data Availability

All data relevant to this research are included within the manuscript or supplied as Supplementary Materials. The 16S rDNA bacterial gene sequencing datasets and mass spectrometry data generated during this investigation have been submitted to the NCBI SRA database (accession number PRJNA1182096). All datasets from this study can be accessed upon reasonable request.

## Supplementary Materials

The following supporting information can be downloaded at: https://www.mdpi.com/article/10.3390/ijms27198824/s1.
