# Supplementary material for: Tangerine Peel Polysaccharide Alleviates High-Fat Diet-Induced Renal Lipotoxicity in Association with Gut Microbiota Remodeling in Mice
Source: Int J Mol Sci. 2026 Oct 2;27(19):8824. doi: 10.3390/ijms27198824 (PMC13664836; doi:10.3390/ijms27198824)
Supplement: Supplementary file 1 [file ijms-27-08824-s001.zip › ijms-4493699-supplementary.pdf]

Supplementary Materials

Tangerine Peel Polysaccharide Alleviates High-Fat Diet-Induced Renal Lipotoxicity in Association with Gut Microbiota Remodeling in Mice

Jingqiu Ma [1,†], Xiuyuan Wu [2,†], Chunbo Wu [2], and Rihui Wu [2,*]

[1] College of Food Science and Engineering, Lingnan Normal University, Zhanjiang 524048, China

[2] School of Pharmacy and Food Engineering, Wuyi University, Jiangmen 529020, China

* Correspondence: j002666@wyu.edu.cn

[†] These authors contributed equally to this study.

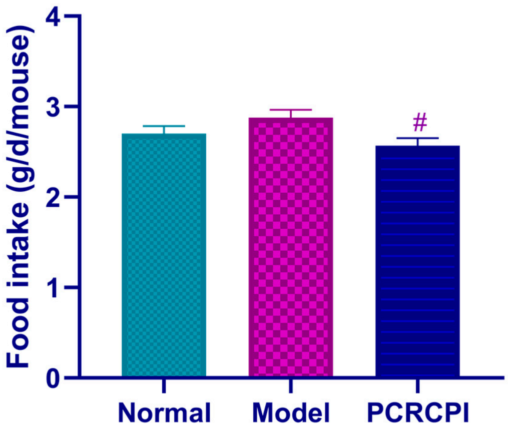

Figure S1. Daily food intake of mice treated with PCRCPI. Data are presented as mean ± SEM (n = 8 per group). # $p < 0.05$ versus the Model group.

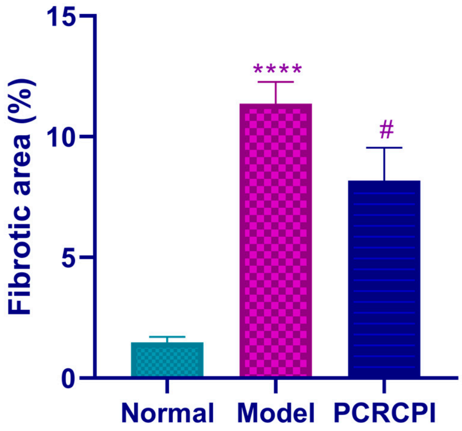

Figure S2. Quantification of Sirius Red staining for renal fibrosis. Sirius Red-positive areas (%) was measured using ImageJ. Data are shown as mean ± SEM (*n* = 8). **** $p < 0.0001$ vs. Normal group; $^{\#}$ $p < 0.05$ vs. Model group.

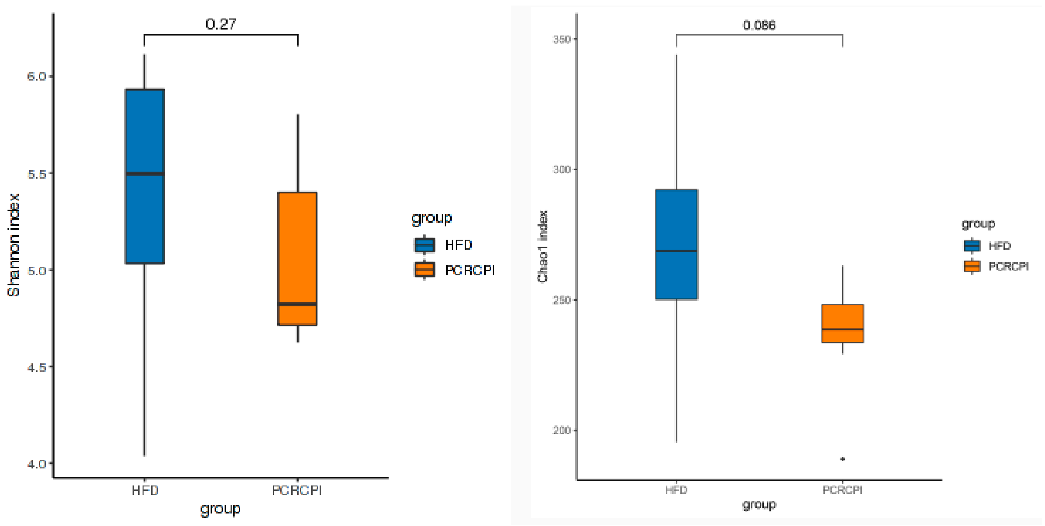

Figure S3. Alpha diversity of gut microbiota was assessed using the Chao1 and Shannon indices.

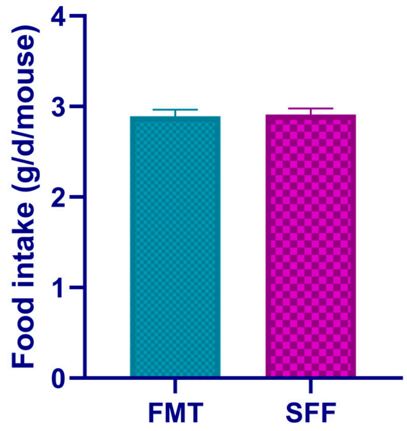

Figure S4. Daily food intake of recipient mice.

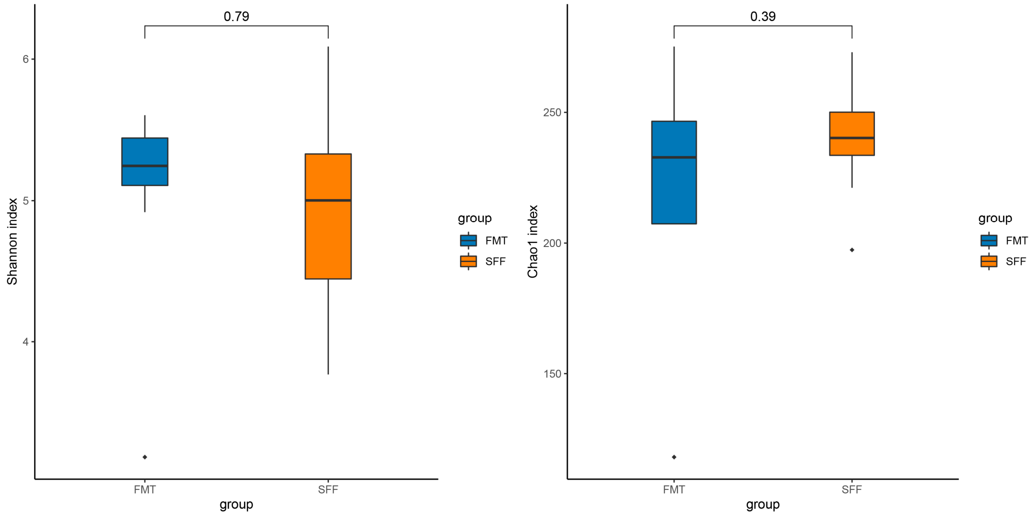

Figure S5. Alpha diversity of gut microbiota of recipients was assessed using the Chao1 and Shannon indices.

Supplementary Table S1. Primer sequences used in this study.

**Genes**	**Forward (5'-3' Sequence)**	**Reverse (5'-3' Sequence)**
ACL	ATGCCAAGACCATCCTCTCACT	TCTCACAATGCCCTTGAAGGT
Mogat1	TGGTGCCAGTTTGGTTCCAG	TGCTCTGAGGTCGGGTTC
α-SMA	AGCCAAGCACTGTCAGGAATC	GTCACCCACGTAGCTGTCTT
TGF-β1	TGCTAATGGTGGACCGCAA	CACTGCTTCCCGAATGTCTGA
Nphs1	ACCTGTATGACGAGGTGGAGAG	TCGTGAAGAGTCTCACACCAG
IL-1β	TGAAAGCTCTCCACCTCAATGG	CTTCTTTGGGTATTGCTTGGG
GDF-15	CAACCAGAGCTGGGAAGATTCG	CCCGAGAGAGATACGCAGGTGC
CCL2	ATGCCAAGACCATCCTCTCACT	TCTCACAATGCCCTTGAAGGT
β-actin	GGCTGTATTCCCCTCCATCG	CCAGTTGGTAACAATGCCATGT
